# Cross-Linked Polyacrylic-Based Hydrogel Polymer Electrolytes for Flexible Supercapacitors

**DOI:** 10.3390/polym16060800

**Published:** 2024-03-13

**Authors:** Lanxin Shi, Pengfei Jiang, Pengxue Zhang, Nannan Duan, Qi Liu, Chuanli Qin

**Affiliations:** School of Chemistry and Materials Science, Heilongjiang University, Harbin 150080, China; 2018027@hlju.edu.cn (L.S.); jpf848185@163.com (P.J.); zhangpengxue11@163.com (P.Z.); duannannan110@163.com (N.D.); 15663710098@163.com (Q.L.)

**Keywords:** cross-linked hydrogel polymer electrolyte, flexible supercapacitor, acrylic copolymer, acrylamide, N-methylolacrylamide

## Abstract

Hydrogel polymer electrolytes (GPEs), as an important component of flexible energy storage devices, have gradually received wide attention compared with traditional liquid electrolytes due to their advantages of good mechanical, bending, and safety properties. In this paper, two cross-linked GPEs of poly(acrylic acid-co-acrylamide) or poly(acrylic acid-co-N-methylolacrylamide) with NaNO_3_ aqueous solution (P(AA-co-AM)/NaNO_3_ or P(AA-co-HAM)/NaNO_3_) were successfully prepared using radical polymerization, respectively, using acrylic acid (AA) as the monomer, N-methylolacrylamide (HAM) or acrylamide (AM) as the comonomer, and N, N-methylenebisacrylamide (MBAA) as the cross-linking agent. We investigated the morphology, glass transition temperature (*T*_g_), ionic conductivities, mechanical properties, and thermal stabilities of the two GPEs. By comparison, P(AA-co-HAM)/NaNO_3_ GPE exhibits a higher ionic conductivity of 2.00 × 10^−2^ S/cm, lower *T*_g_ of 152 °C, and appropriate mechanical properties, which are attributed to the hydrogen bonding between the -COOH and -OH, and moderate cross-linking. The flexible symmetrical supercapacitors were assembled with the two GPEs and two identical activated carbon electrodes, respectively. The results show that the flexible supercapacitor with P(AA-co-HAM)/NaNO_3_ GPE shows good electrochemical performance with a specific capacitance of 63.9 F g^−1^ at a current density of 0.2 A g^−1^ and a capacitance retention of 89.4% after 3000 charge–discharge cycles. Our results provide a simple and practical design strategy of GPEs for flexible supercapacitors with wide application prospects.

## 1. Introduction

Over the past decade, with the rapid development of portable electronics and electric vehicles, batteries and supercapacitors as electrical energy storage devices have received increasing attention from both academia and industry [1]. Batteries as traditional energy storage devices own high energy density, but they have low power outputting capability [2,3,4,5,6]. In contrast, supercapacitors are promising for applications because of their higher power density and efficiency, as well as higher cycle durability, along with faster charging and discharging capability and lower cost [7,8,9]. In supercapacitors, electrolytes play a key role in determining the electrochemical and mechanical properties of supercapacitors. Especially, some supercapacitors require flexibility and resistance to deformation to meet the demands of applications under different conditions [10,11,12,13,14]. However, conventional liquid electrolytes have serious limitations in that there is the risk of accidental leakage when the supercapacitor is repeatedly bent or compressed. Therefore, considering the high ionic conductivity, tunable mechanical property, flexibility, and dimensional stability, the hydrogel polymer electrolytes (GPEs) are the ideal candidate for flexible all-solid-state supercapacitors [15,16]. Nowadays, many efforts have been devoted to the development of GPEs as an alternative to liquid electrolytes, aiming to enable supercapacitors to be used under more demanding mechanical conditions [17,18].

GPEs usually consist of 3D networks with physically or chemically cross-linked polymer chains, and the internal space is filled with an aqueous electrolyte making it an ionic conductor [19,20]. Compared with traditional electrolytes, GPEs can hold the aqueous electrolyte through the physically or chemically cross-linked polymer chains in terms of disposal, leakage, or during operation at high temperatures, so they own environmental or safety advantages [21,22]. The mesh size of polymer networks is larger than the size of ions and water molecules from the electrolyte, thus allowing the electrolyte in the hydrogel to maintain the same chemical and physical properties as in the electrolyte solution [23]. The main function of GPEs is not only to serve as an ionic conducting medium, but also to act as an isolator. Up to now, researchers have designed and prepared many different structures of GPEs for supercapacitors. Liu et al. prepared an all-natural GPE membrane by a simple two-step method, in which chitosan, sodium carboxymethylcellulose, and tannic acid were physically cross-linked through hydrogen bonding interactions. As a natural polyphenol with strong antioxidant and antibacterial properties derived from plants, tannic acid contains a large amount of active -OH on the molecular chain, which can form hydrogen bonds with -OH on polymer chains. Therefore, tannic acid is chosen as a physical cross-linking agent to interact with two natural polysaccharides at the same time, thereby effectively improving the mechanical properties of the hydrogel [24]. Yang et al. synthesized a modified supramolecular carboxylated chitosan (CYCTS-g-PAM-Li_2_SO_4_) GPE through a radical graft copolymerization of acrylamide monomers and chemical cross-linking. Compared with PAM-based GPE (PAM-Li_2_SO_4_), the modified CYCTS-g-PAM-Li_2_SO_4_ GPE exhibits better performance [25]. Kamarulazam et al. prepared polyacrylamide GPEs by the radical polymerization and physical cross-linking. The electrochemical impedance spectroscopy (EIS) revealed that GPE with 40 wt.% lithium acetate as a source of mobile ions achieved the highest ionic conductivity of 9.45 × 10^−3^ S cm^−1^ [26]. Through previous research, it is found that high ionic conductivity, appropriate cross-linking, and mechanical properties of GPEs are very critical for applications in the field of supercapacitors.

The acrylic monomer has the -COOH, and when it copolymerizes with some monomers with the -OH or -NH_2_, the copolymers own a large amount of hydrogen bonding and enable their GPEs to exhibit stable shape and good mechanical properties. Furthermore, their GPEs with the physical cross-linking structure exhibits porous microstructure, which is favorable for ion transport. Especially, adjusting the cross-linking degree of GPEs could reduce the glass transition temperature (*T*_g_), in favor of chain segment mobility, and thus high ionic conductivity. Therefore, the cross-linked GPEs with hydrogen bonding interactions, low *T*_g_, and porous structure owns high ionic conductivity and moderate mechanical properties, contributing to the improved electrochemical properties of their corresponding flexible supercapacitors.

In this work, we successfully prepared two cross-linked GPEs, which are comprised of poly(acrylic acid-co-acrylamide) or poly(acrylic acid-co-N-methylolacrylamide) with NaNO_3_ aqueous solution (P(AA-co-AM)/NaNO_3_ or P(AA-co-HAM)/NaNO_3_) by radical polymerization of acrylic acid (AA) as the monomer, N-methylolacrylamide (HAM) or acrylamide (AM) as the comonomer, and N, N-methylenebisacrylamide (MBAA) as the cross-linking agent. The morphology, glass transition temperature (*T*_g_), ionic conductivities, mechanical properties, and thermal stabilities of the two GPEs were mainly investigated and compared. The -COOH in the AA repeating unit can form hydrogen bonds with -NH_2_ in the AM repeating unit and -OH in the HAM repeating unit, respectively. Compared to P(AA-co-AM)/NaNO_3_ GPE, P(AA-co-HAM)/NaNO_3_ GPE exhibits a lower *T*_g_ with stronger mobility of chain segments, which facilitates ionic migration and thus a higher ionic conductivity, and appropriate mechanical property, which is attributed to the hydrogen bonding between the -COOH and -OH, and moderate cross-linking. Our work focuses on the effect of hydrogen bonding interactions on the *T*_g_, ionic conductivities and mechanical properties of the GPEs and electrochemical performance of the assembled supercapacitors. The synthesized P(AA-co-HAM)/NaNO_3_ GPE has a wide range of application prospects for flexible, wearable, and smart energy storage devices.

## 2. Methods

### 2.1. Materials

Acrylic acid (AA, 99%, Fuchen Tianjin Chemical Reagent Co., Ltd., Tianjin, China), N-methylolacrylamide (HAM, 97%, Rhön Reagent Co., Ltd., Shanghai, China), acrylamide (AM, 98%, Rhön Reagent Co., Ltd., Shanghai, China), N, N-methylenebisacrylamide (MBAA, 98%, Shanghai Diba Bio-technology Co., Ltd., Shanghai, China), ammonium persulfate (APS, 98%, Fuchen Tianjin Chemical Reagent Co., Ltd., Tianjin, China), sulfuric acid (10%, Tianjin Fuyu Fine Chemical Co., Ltd., Tianjin, China), hydrochloric acid (10%, Tianjin Fuyu Fine Chemical Co., Ltd., Tianjin, China), NaNO_3_ solution (3 mol/L, Harbin Kemis Technology Co., Ltd., Harbin, China), polytetrafluoroethylene mold (Harbin Kemis Technology Co., Ltd., Harbin, China), activated carbon (AC, 99%, Harbin Kemis Technology Co., Ltd., Harbin, China), graphite (97%, Harbin Kemis Technology Co., Ltd., Harbin, China), polyvinylidene fluoride (PVDF, 60%, Shanghai Aladdin Biochemical Technology Co., Ltd., Shanghai, China), carbon cloth (Suzhou Keshenghe Co., Ltd., Suzhou, China), and PET membrane (Shanghai Xinya Purification Device Factory Co., Ltd., Shanghai, China).

### 2.2. Synthesis

#### 2.2.1. Synthesis of P(AA-co-HAM)/NaNO_3_ GPE

**Figure 1:**
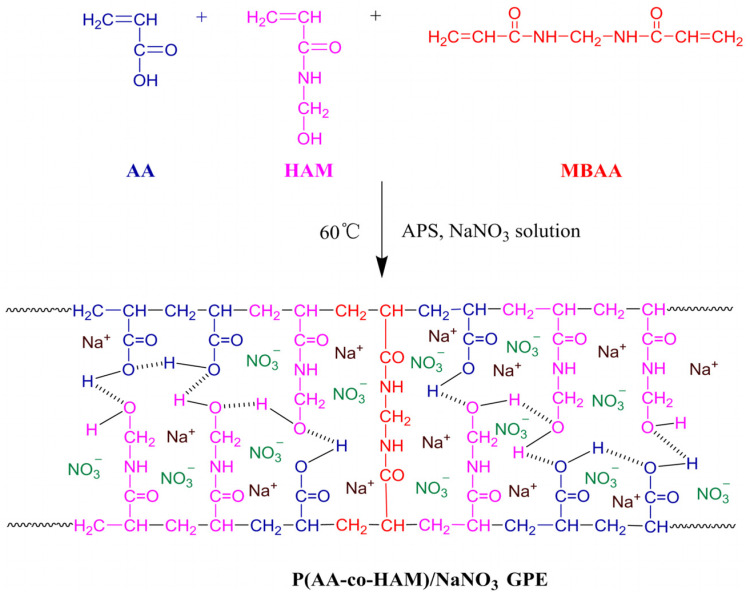
The synthesis route of P(AA-co-HAM)/NaNO_3_ GPE.

An amount of 1 mL of AA, 1.01 g of HAM, 20 mg of MBAA, 30 mg of APS, and 4 mL of NaNO_3_ solution (3 mol/L) were sequentially added to a 10 mL beaker, mixed, and stirred for 2 h at room temperature. The obtained solution was poured into a PTFE mold, placed in a vacuum oven, and held at 60 °C for 1.5 h. It was then cooled to room temperature and demolded to obtain a solid GPE (40 mm × 20 mm × 5 mm). The synthesis route of P(AA-co-HAM)/NaNO_3_ GPE is shown in Figure 1.

#### 2.2.2. Synthesis of P(AA-co-AM)/NaNO_3_ GPE

**Figure 2:**
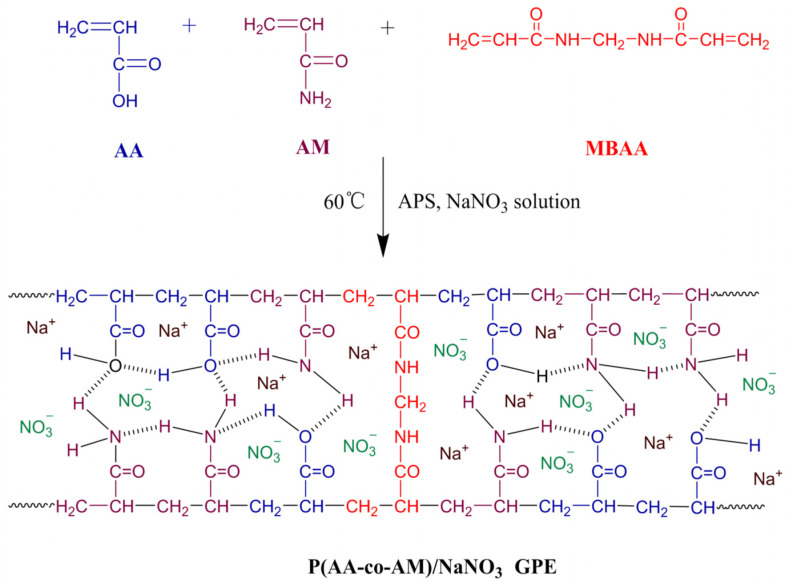
The synthesis route of P(AA-co-AM)/NaNO_3_ GPE.

0.71 g of AM were added instead of 1.01 g of HAM. The other regents and synthesis process are the same as those for the preparation of P(AA-co-HAM)/NaNO_3_ GPE. The synthesis route of P(AA-co-AM)/NaNO_3_ GPE is shown in Figure 2.

#### 2.2.3. Preparation of Electrodes and Assembly of Supercapacitors

The electrode was prepared as shown below. The carbon cloth was first soaked in a 3:1 mixture of 10% HNO_3_ and 10% H_2_SO_4_ for 12 h for hydrophilic treatment, washed with deionized water three times before being ultrasonically cleaned with deionized water for 30 min, and then dried to remove the residual HNO_3_ and H_2_SO_4_ from the carbon cloth. Appropriate amounts of activated carbon (AC), graphite, PVDF binder with the mass ratio of 8:1:1, and an adequate amount of ethanol were added to a beaker, and, after stirring for 30 min, a well-mixed electrode slurry was obtained. The carbon cloth was cut to a suitable size (10 mm × 30 mm), and the slurry was uniformly coated on the cleaned carbon cloth (the loaded active substance was about 5 mg), placed in a vacuum drying oven at 80 °C for 24 h, and then cooled to room temperature to obtain an activated carbon electrode.

**Figure 3:**
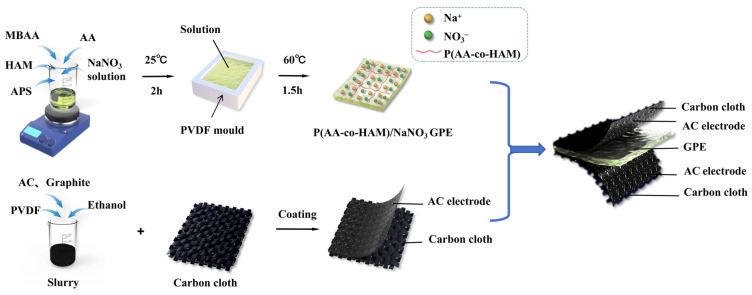
Schematic representation of the preparation process of P(AA-co-HAM)/NaNO_3_ GPE, activated carbon electrode, and the assembly of a supercapacitor.

The prepared GPE was sandwiched between two activated carbon electrodes, packaged with PET film, and then sealed using hot melt adhesive to obtain a supercapacitor device. Figure 3 shows the preparation process of P(AA-co-HAM)/NaNO_3_ GPE, activated carbon electrode, and the assembly of a supercapacitor.

### 2.3. Characterization

Fourier transform infrared (FTIR) spectra of samples were collected with a spectrometer (Bruker Tyskland, Equiox 55, Bruker, Germany) in the range of 400–4000 cm^−1^ with a resolution of 6 cm^−1^ for 32 scans of GPEs using the KBr pellet technique. A integrated thermal analyzer (Beijing Hengjiu, HCT-4, Beijing, China) was used to assess *T*_g_ and thermal stability of GEPs from room temperature to 400 °C under N_2_ atmosphere at a heating rate of 5 °C/min. A scanning electron microscope (SEM, Zeiss, Gemini SEM 300, Shanghai, China) was used to investigate the morphology and microstructure of GPEs. The mechanical properties of GPEs (30 mm × 15 mm × 2 mm) were tested at a constant tensile speed of 2 mm/min at room temperature using a tensile tester (Wenteng Testing Instruments, XLW (PC)-500N, Jinan, China). The ionic conductivity of GPEs was determined at room temperature using a four-probe conductivity meter (Guangzhou Four Probe Technology Co., RTS-9, Guangzhou, China) [27,28]. The electrochemical performance of supercapacitors were investigated by the following methods. Cyclic voltammetry (CV) tests were performed by a computerized electroanalytical system (Tianjin Lannico, LK98B II, Tianjin, China) at room temperature in the potential window range of 0 to 1 V at different scan rates from 10 mV/s to 100 mV/s. Charge–discharge (GCD) measurements were performed using an electrochemical workstation (LAND Wuhan Jinnuo, CT2001A, Wuhan, China) at current densities from 0.2 A g^−1^ to 1.0 A g^−1^. EIS measurements were performed over a frequency range of 0.01 Hz to 100 kHz with an amplitude of 10 mV−0.1 V at the open-circuit potential of −0.1 V with the electrochemical workstation (Shanghai Chenhua, CHI660E-B19775, Shanghai, China). The specific capacitance (*C*_g_, F g^−1^) of supercapacitors was calculated based on GCD curves with the following Equation (1).

(1) $$C_{g}=\frac{I\times \Delta t}{m\times \Delta v}$$

The specific energy (*E*, Wh kg^−1^) and specific power (*P*, W kg^−1^) of supercapacitors were calculated with the following Equation (2).

(2) $$E=\frac{C_{g}\times \Delta v^{2}}{7.2}$$

(3) $$P=\frac{E\times 3600}{\Delta t}$$

where *I* (A) is the current density, *m* is the total mass of active substances on both electrodes (g), Δt is the discharge time (s), and Δv is the voltage range after the IR drop during the discharge process (V) [29].

## 3. Results and Discussion

### 3.1. Infrared Analysis

**Figure 4:**
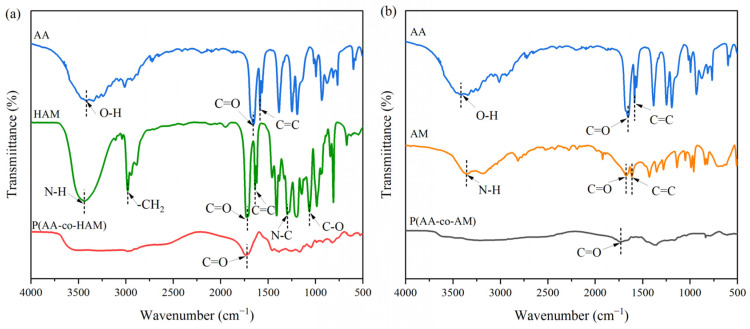
FTIR spectra of (**a**) AA, HAM, and P(AA-co-HAM), and (**b**) AA, AM, and P(AA-co-AM).

The compositions of AA, HAM, and P(AA-co-HAM) were studied using FTIR spectra, as shown in Figure 4a. The spectrum of AA shows three main significant vibration peaks in the range of 500–4000 cm^−1^: stretching vibration peak of O-H at 3430 cm^−1^, the stretching vibration peak of C=O and C=C at 1695 and 1635 cm^−1^ in the -COOH, respectively [30,31,32]. The main characteristic peaks of HAM appear at 3443 cm^−1^ (N-H asymmetric stretching vibration), 2935 cm^−1^ (-CH_2_ asymmetric stretching vibration), 1737 cm^−1^ (C=O stretching vibration), 1640 cm^−1^ (C=C stretching vibration), 1274 cm^−1^ (N-C stretching vibration), and 1021 cm^−1^ (C-O stretching vibration) [30,32,33]. In contrast, P(AA-co-HAM) show the characteristic peaks of AA and HAM, and the C=C stretching vibration peaks of AA and HAM at 1635 and 1640 cm^−1^ disappear after P(AA-co-HAM) was synthesized, confirming the synthesis of P(AA-co-HAM)/NaNO_3_ GPE. Figure 4b shows the FTIR spectra of AA, AM, and P(AA-co-AM). The main characteristic peaks of AM appear in the regions of 3100 cm^−1^ to 3500 cm^−1^ (N-H band), 1650 cm^−1^ (C=O stretching vibration), and 1618 cm^−1^ (C=C stretching vibration) [32,34]. Similarly, P(AA-co-AM) show the characteristic peaks of AA and AM, and the C=C stretching vibration peaks of AA and AM at 1635 and 1618 cm^−1^ disappear after P(AA-co-AM) was synthesized, confirming the synthesis of P(AA-co-AM)/NaNO_3_ GPE.

### 3.2. Mechanical Properties Analysis

**Figure 5:**
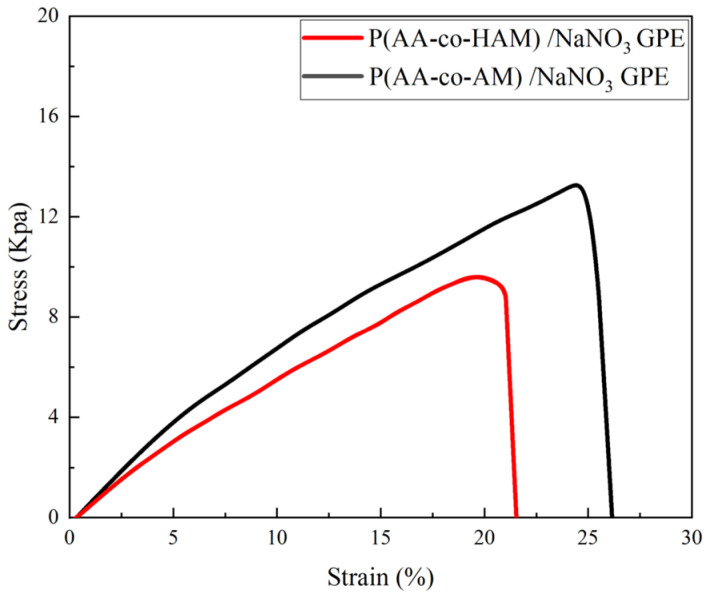
Stress–strain curves of P(AA-co-HAM)/NaNO_3_ GPE and P(AA-co-AM)/NaNO_3_ GPE.

The mechanical strength of GPEs is the essential property for the application of flexible supercapacitors. The stress–strain curves of P(AA-co-HAM)/NaNO_3_ GPE and P(AA-co-AM)/NaNO_3_ GPE are presented in Figure 5. From the curves, it can be seen that both GPEs exhibits the characteristics of elastomers. The maximum stress of P(AA-co-HAM)/NaNO_3_ GPE and P(AA-co-AM)/NaNO_3_ GPE are 9.6 KPa and 13.9 KPa, respectively. The fracture strain of P(AA-co-AM)/NaNO_3_ GPE and P(AA-co-HAM)/NaNO_3_ GPE are 26% and 20%, respectively. By comparison, P(AA-co-HAM)/NaNO_3_ GPE shows slightly lower fracture stress and strain than P(AA-co-AM)/NaNO_3_ GPE, which could be due to the fact that before polymerization -COOH in AA monomer can form stronger hydrogen bonding with -OH in HAM monomer than -NH_2_ in AM monomer, resulting in the slightly lower cross-linking degree and mechanical properties of synthesized P(AA-co-HAM)/NaNO_3_ GPE than those of P(AA-co-AM)/NaNO_3_ GPE.

### 3.3. Thermal Stability Analysis

*T*_g_ and thermal stabilities of GPEs are very important for their applications. The lower *T*_g_ of GPEs means that it has more flexible chains, which helps the transport of electrolyte ions. So we carried out the DSC and TG measurements to determine the *T*_g_ and thermal stability properties of the synthesized GPE.

**Figure 6:**
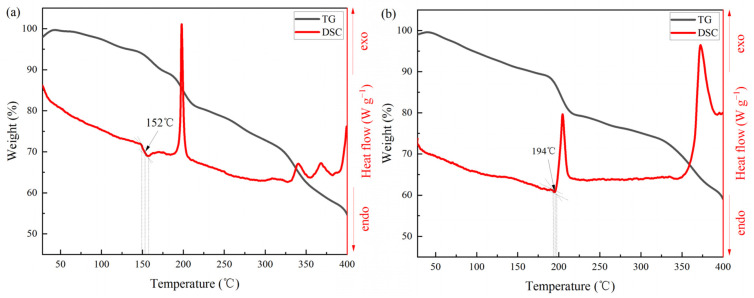
DSC and TG curves of (**a**) P(AA-co-HAM)/NaNO_3_ GPE and (**b**) P(AA-co-AM)/NaNO_3_ GPE.

Figure 6 shows DSC and TG curves of P(AA-co-HAM)/NaNO_3_ GPE and P(AA-co-AM)/NaNO_3_ GPE. It can be clearly seen that the *T*_g_ of P(AA-co-HAM)/NaNO_3_ GPE and P(AA-co-AM)/NaNO_3_ GPE are ~152 °C and ~194 °C, respectively. By comparison, P(AA-co-HAM)/NaNO_3_ GPE shows a lower *T*_g_ with the promoted movement of polymer segments and increased flexibility, which greatly contributes to the transport of electrolyte ions, thus endows its supercapacitor superior electrochemical performance. It also can be seen that there is no significant difference in the thermal stabilities of the two GPEs. There are mainly three thermal decomposition steps. An initial weight loss of 5–10 wt.% in the temperature range of 50–150 °C is due to the evaporation of absorbed water. In the temperature range of 150–250 °C, the two GPEs show significant weight loss of 10–25 wt.%, which is due to the decomposition of groups in the polymer backbone. A rapid weight loss of 25–50 wt.% takes place over the temperature range of 250–400 °C, which is attributed to the degradation of polymer chains.

### 3.4. Ionic Conductivity Analysis

The ionic conductivities of P(AA-co-HAM)/NaNO_3_ GPE and P(AA-co-AM)/NaNO_3_ GPE at room temperature for different periods (0, 7, and 14 days) are given in Table 1. At 0 day, 7 days, and 14 days, the conductivities of P(AA-co-HAM)/NaNO_3_ GPE are 2.00 × 10^−2^ S/cm, 1.96 × 10^−2^ S/cm and 1.82 × 10^−2^ S/cm, respectively, while those of P(AA-co-AM)/NaNO_3_ GPE are 6.13 × 10^−3^ S/cm, 5.95 × 10^−3^ S/cm, and 5.56 × 10^−3^ S/cm, respectively. Both the two GPEs display high ionic conductivities, and with the increase of time, their conductivities do not decay significantly, indicating that they have good electrolyte retention capacities. By comparison, the ionic conductivities of the prepared GPEs, especially that of P(AA-co-HAM)/NaNO_3_ GPE, are higher than those of the reported GPEs (as shown in Table S1). It is due to the fact that P(AA-co-HAM)/NaNO_3_ GPE has lower *T*_g_ (as shown in Figure 6a) and richer pore structure (as shown in Figure S1), which are conducive to the migration and rapid diffusion of electrolyte ions [35,36]. It is expected that the supercapacitor with P(AA-co-HAM)/NaNO_3_ GPE will exhibit a higher electrochemical properties.

### 3.5. Electrochemical Performances of Supercapacitors

To further investigate the electrochemical properties of the prepared GPEs, we assembled flexible symmetric supercapacitors using P(AA-co-HAM)/NaNO_3_ GPE or P(AA-co-AM)/NaNO_3_ GPE and two identical activated carbon electrodes (AC//P(AA-co-HAM)/NaNO_3_//AC and AC//P(AA-co-AM)/NaNO_3_//AC), respectively.

**Figure 7:**
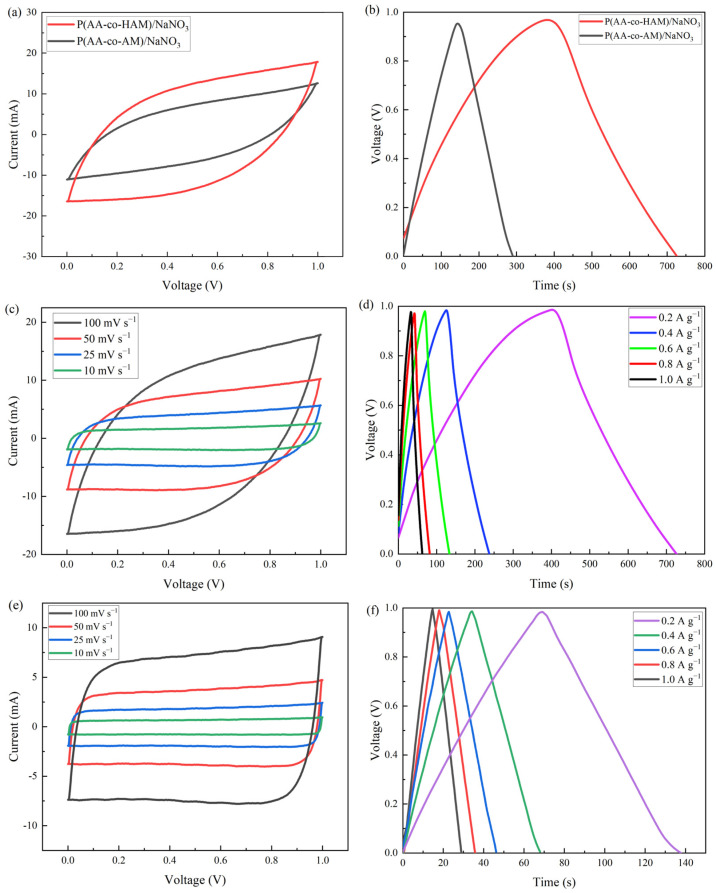
Electrochemical performance of AC-based symmetrical supercapacitors with different GPEs: (**a**) CV curves at 100 mV s^−1^ and (**b**) GCD curves at 0.2 A g^−1^; electrochemical performance of AC-based symmetrical supercapacitors with P(AA-co-HAM)/NaNO_3_ GPE: (**c**) CV curves at different scan rates, and (**d**) GCD curves at different current densities; electrochemical performance of conventional aqueous supercapacitor with NaNO_3_ aqueous solution: (**e**) CV curves at different scan rates, and (**f**) GCD curves at different current densities.

Their electrochemical properties were investigated. The CV curves of the two AC-based symmetrical supercapacitors with different GPEs at 100 mV s^−1^ are given in Figure 7a. It can be found that the CV curves of two supercapacitors show rectangular shapes under the voltage window from 0 V to 1.0 V, indicating good double layer capacitance behavior. By comparison, the AC//P(AA-co-HAM)/NaNO_3_//AC has a rectangular shape with the largest closed area, showing the best capacitive behavior. In Figure 7b, the GCD curves of the two supercapacitors with different GPEs show the symmetrical triangular shapes at 0.2 A g^−1^. By comparison, the charge–discharge curve of AC//P(AA-co-HAM)/NaNO_3_//AC has the longest discharge time. Based on the Equation (1), the *C*_g_ value of AC//P(AA-co-HAM)/NaNO_3_//AC is 63.9 F g^−1^ at 0.2 A g^−1^, which is higher than that of AC//P(AA-co-AM)/NaNO_3_//AC (50.1 F g^−1^). Moreover, as shown in Figure 7c and Figure S2a, even at a scan rate of 100 mV s^−1^, the CV curves of AC//P(AA-co-HAM)/NaNO_3_//AC and AC//P(AA-co-AM)/NaNO_3_//AC show no distortion, implying their good rate performance [37,38]. Figure 7d and Figure S2b show their GCD curves from 0.2 A g^−1^ to 1 A g^−1^. As the current density increases, it can be seen that their GCD curves remain symmetrically triangular, indicating typical reversible charging and discharging behavior [39], further confirming its good rate performance. In order to compare the performance of the GPEs and conventional aqueous electrolytes, the conventional aqueous supercapacitor was assembled with 3 mol/L NaNO_3_ aqueous solution as the electrolyte. As can be seen from its CV curves (Figure 7e) and GCD curve (Figure 7f), the conventional aqueous supercapacitor also shows a rectangular shape and symmetrical triangle, and its calculated *C*_g_ value is 34.8 F g^−1^ at 0.2 A g^−1^, obviously lower than those of the two supercapacitors with GPEs. It indicates that the synthesized GPEs, especially P(AA-co-HAM)/NaNO_3_ GPE, are superior to conventional aqueous electrolytes.

**Figure 8:**
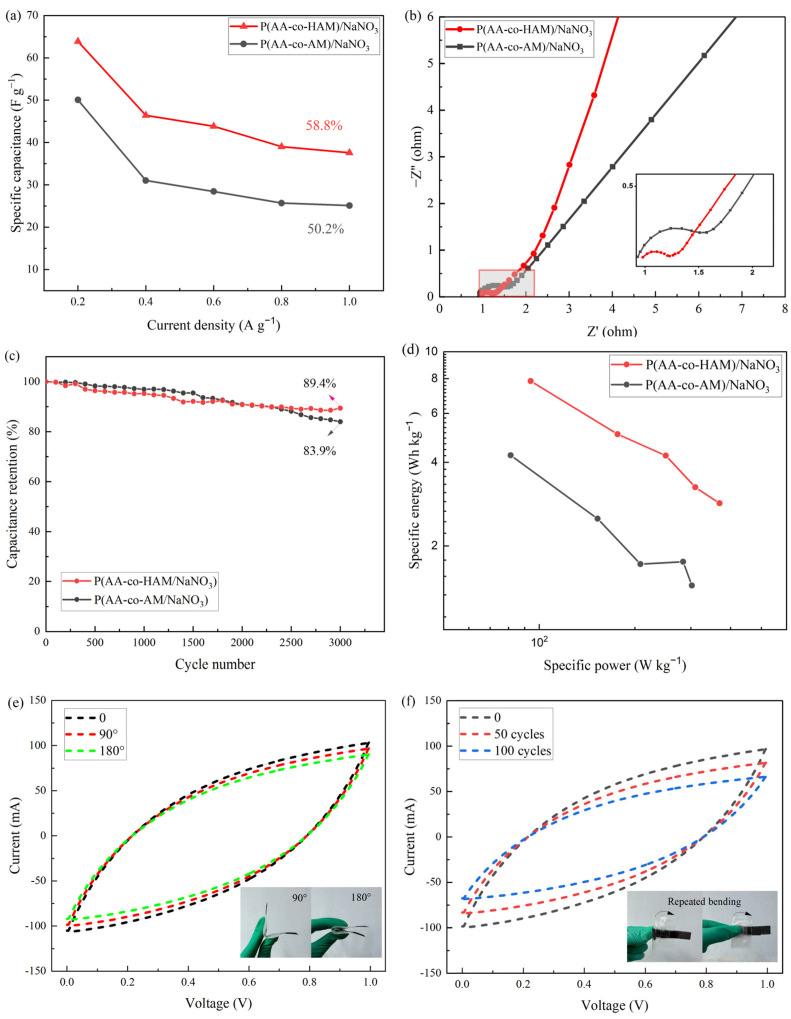
Electrochemical performance of AC-based symmetrical supercapacitors with different GPEs: (**a**) specific capacitances at different current densities, (**b**) Nyquist plots, and (**c**) cycling stability measured at 1 A g^−1^ for 3000 cycles; (**d**) Ragone plot; CV curves of AC-based symmetrical supercapacitors with P(AA-co-HAM)/NaNO_3_ GPE; (**e**) with different bending angles; and (**f**) with different bending cycles, inset: photos under the bending conditions.

The *C*_g_ values of two supercapacitors with different GPEs at different current densities are shown in Figure 8a. When the current density increases from 0.2 A g^−1^ to 1.0 A g^−1^, the *C*_g_ value of AC//P(AA-co-HAM)/NaNO_3_//AC decreases from 63.9 F g^−1^ to 37.6 F g^−1^, showing a better rate capability with a capacitance retention of 58.8% than AC//P(AA-co-AM)/NaNO_3_//AC (50.2%). Figure 8b shows Nyquist plots for two symmetrical supercapacitors with different GPEs. Both diagrams include a semicircle and sloping line. In the low-frequency region, the Nyquist plot of AC//P(AA-co-HAM)/NaNO_3_//AC is closer to the imaginary axis than that of AC//P(AA-co-AM)/NaNO_3_//AC, which indicates that AC//P(AA-co-HAM)/NaNO_3_//AC has the faster diffusion rate of electrolyte ions. Furthermore, it is clear at high frequencies the semicircular diameter of AC//P(AA-co-HAM)/NaNO_3_//AC is smaller than that of AC//P(AA-co-AM)/NaNO_3_//AC, which indicates its smaller charge transfer resistance, contributing to its better capacitive performance. The cycling stabilities of the two symmetrical supercapacitors with different GPEs are given in Figure 8c. After 3000 charge–discharge cycles, the capacitance retention of AC//P(AA-co-HAM)/NaNO_3_//AC is 89.4%, which is higher than AC//P(AA-co-AM)/NaNO_3_//AC (83.9%), showing its better cycling stability. It is attributed to the cross-linking structure stability of P(AA-co-HAM)/NaNO_3_ GPE because of the stronger hydrogen bonding interactions between -COOH and -OH in P(AA-co-HAM)/NaNO_3_ GPE than those between -COOH and -NH_2_ in P(AA-co-AM)/NaNO_3_ GPE. Figure 8d shows Ragone plots of the two supercapacitors with different GPEs. The AC//P(AA-co-HAM)/NaNO_3_//AC achieves a specific energy of 7.83 Wh kg^−1^ at a specific power of 93.98 W kg^−1^, which is superior to that of AC//P(AA-co-AM)/NaNO_3_//AC (a specific energy of 4.24 Wh kg^−1^ at a specific power of 81.30 W kg^−1^). Based on the above analysis, it is concluded that the synthesized P(AA-co-HAM)/NaNO_3_ GPE is superior to P(AA-co-AM)/NaNO_3_ GPE and conventional aqueous electrolyte for supercapacitor applications. To further investigate the mechanical flexibility of the optimal AC//P(AA-co-HAM)/NaNO_3_//AC, its CV tests were performed under various bending conditions. As shown in Figure 8e,f, at different bending angles (90° and 180°) and repeat bending cycles from 0 to 100 cycles, all the CV curves display a similar shape and area, indicating that the bending conditions do not obviously influence the electrochemical performance and the synthesized P(AA-co-HAM)/NaNO_3_ GPE is suitable for flexible supercapacitors with high performance.

## 4. Conclusions

In conclusion, two cross-linked hydrogel polymer electrolytes of P(AA-co-HAM)/NaNO_3_ and P(AA-co-AM)/NaNO_3_ were successfully developed by radical polymerization at room temperature. By comparison, due to stronger hydrogen bonding between the -COOH and -OH, as well as moderate cross-linking, P(AA-co-HAM)/NaNO_3_ hydrogel polymer electrolyte exhibits a higher ionic conductivity of 2.00 × 10^−2^ S/cm, lower glass transition temperature of 152 °C, and appropriate mechanical properties. The assembled AC//P(AA-co-HAM)/NaNO_3_//AC symmetrical supercapacitor show the specific capacitance of 63.9 F g^−1^ at 0.2 A g^−1^, capacitance retention of 89.4% after 3000 change–discharge cycles, and the power density of 93.98 W kg^−1^ at an energy density of 7.83 Wh kg^−1^, which is obviously higher than those of supercapacitors assembled with P(AA-co-AM)/NaNO_3_ GPE and a conventional aqueous electrolyte. Thus, P(AA-co-HAM)/NaNO_3_ GPE would be a promising electrolyte for flexible and high-performance supercapacitor devices.

## Figures and Tables

**Table 1:** Ionic conductivity of P(AA-co-HAM)/NaNO_3_ GPE and P(AA-co-AM)/NaNO_3_ GPE at room temperature for different periods.

GPE	Resistivity (kΩ·cm)	Ionic Conductivity (S/cm)
P(AA-co-HAM/NaNO_3_) (0 day)	0.050	2.00 × 10^−2^
P(AA-co-HAM/NaNO_3_) (7 days)	0.051	1.96 × 10^−2^
P(AA-co-HAM/NaNO_3_) (14 days)	0.055	1.82 × 10^−2^
P(AA-co-AM/NaNO_3_) (0 day)	0.163	6.13 × 10^−3^
P(AA-co-AM/NaNO_3_) (7 days)	0.168	5.95 × 10^−3^
P(AA-co-AM/NaNO_3_) (14 days)	0.180	5.56 × 10^−3^

## Data Availability

The data presented in this study are available on request from the corresponding author.

## Supplementary Materials

The following supporting information can be downloaded at: https://www.mdpi.com/article/10.3390/polym16060800/s1, Figure S1: SEM images of the washed and freeze-dried samples: (a) P(AA-co-HAM) and (b) P(AA-co-AM), Figure S2: Electrochemical performance of AC-based symmetrical supercapacitors with P(AA-co-AM)/NaNO_3_ GPE: (a) CV curves at different scan rates and (b) GCD curves at different current densities, Table S1: Ionic conductivity comparison of our GPEs and some previously reported GPEs. Refs. [40,41,42,43,44,45,46,47] are cited in the supplementary materials.
