# Supplementary material for: Cross-Linked Polyacrylic-Based Hydrogel Polymer Electrolytes for Flexible Supercapacitors
Source: Polymers (Basel). 2024 Mar 13;16(6):800. doi: 10.3390/polym16060800 (PMC10974375; doi:10.3390/polym16060800)
Supplement: Supplementary file 1 [file polymers-16-00800-s001.zip › polymers-2879814-supplementary.pdf]

Supplementary Information

Cross-Linked Polyacrylic-Based Hydrogel Polymer Electrolytes for Flexible Supercapacitors

Lanxin Shi, Pengfei Jiang, Pengxue Zhang, Nannan Duan, Qi Liu and Chuanli Qin *

School of Chemistry and Materials Science, Heilongjiang University, Harbin 150080, China;
2018027@hlju.edu.cn (L.S.); jpf848185@163.com (P.J.); zhangpengxue11@163.com (P.Z.);
duannannan110@163.com (N.D.); 15663710098@163.com (Q.L.)
* Correspondence: qinchuanli@hlju.edu.cn; Tel.: +86-0451-8660-8543

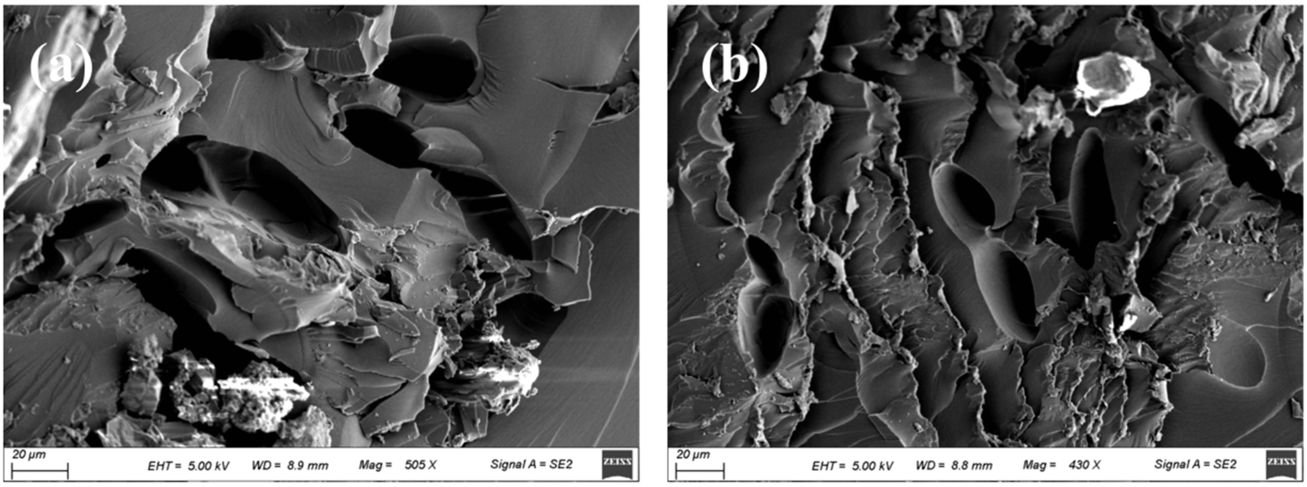

Figure S1. SEM images of the washed and freeze-dried samples: (a) P(AA-co-HAM) and (b) P(AA-co AM).

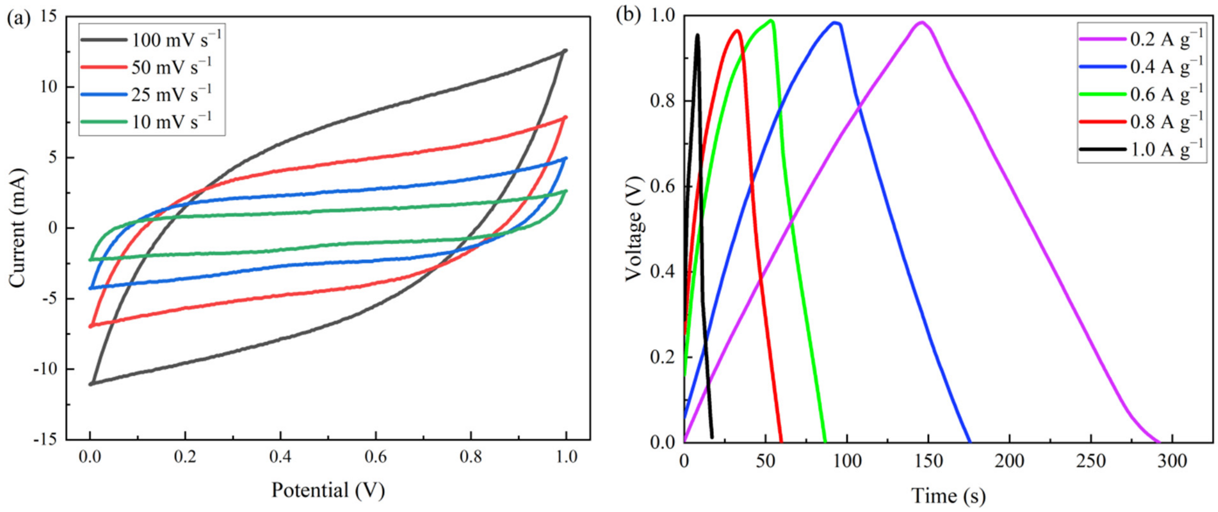

Figure S2. Electrochemical performance of AC-based symmetrical supercapacitors with P(AA-co-AM)/$NaNO_3$ GPE: (a) CV curves at different scan rates and (b) GCD curves at different current densities.

Table S1. Ionic conductivity comparison of our GPEs and some previously reported GPEs

Gel polymer electrolyte	Ionic conductivity (S/cm)	Reference
PVdF(HFP)	4×10^{-5}	[40]
QCS/rGO-PDA/PNIPAm	4.20×10^{-3}	[41]
P(VDF-CO-HFP)	1.62×10^{-3}	[42]
SPI-CO-EGDE	2.79×10^{-3}	[43]
P(VDF-HFP）	2.01×10^{-4}	[44]
PVA:NH_4CH_3COO	2.64×10^{-4}	[45]
PVA-LS-Cl	2.48×10^{-4}	[46]
PLMA-b-POEM	$\sim10^{-5}$	[47]
P(AA-co-HAM)/$NaNO_3$	2.00×10^{-2}	this work
P(AA-co-AM)/$NaNO_3$	6.13×10^{-3}	this work
